# Contributions from active dendritic conductances to the Local Field Potential

**DOI:** 10.1186/1471-2202-16-S1-P203

**Published:** 2015-12-18

**Authors:** Torbjørn V Ness, Michiel WH Remme, Gaute T Einevoll

**Affiliations:** 1 Dept. of Mathematical Sciences and Technology, Norwegian University of Life Sciences, Ås, Norway; 2 Institute for Theoretical Biology, Humboldt University Berlin, Berlin, Germany; 3 Dept. of Physics, University of Oslo, Oslo, Norway

## 

The Local Field Potential (LFP) is typically assumed to mainly stem from synaptic inputs and their subthreshold dendritic processing. The role of active dendritic conductances in shaping the LFP has often been ignored, even though several ion channels are active in the subthreshold voltage regime. Here we use biophysical modeling to investigate the impact of active conductances and their spatial distributions on the LFP in the subthreshold voltage regime.

We used models of neocortical pyramidal neurons with realistic morphologies and systematically varied the spatial distributions of active conductances and synaptic inputs. We linearized the active conductances -- yielding the so-called quasi-active description -- thereby reducing the number of model parameters and highlighting that active currents can be divided into two classes: regenerative and restorative. In terms of the filtering properties of the cell membrane, regenerative conductances amplify low frequencies of the synaptic input current, whereas restorative conductances dampen them.

The low-frequency components of the synaptic input current give the largest contributions to the LFP, due to their larger distance between synaptic currents and associated return currents. Since active conductances can enhance or diminish a neuron's response to low frequencies, they are also expected to affect the LFP.

We indeed found that the effect of active conductances on the single-neuron LFP is strongest for the lowest frequencies (< 10-30 Hz). The impact was most pronounced when 1) the synaptic input is asymmetric (i.e., either basal or apical), 2) the active conductances are distributed non-uniformly with the highest channel densities near the synaptic input, and 3) when the LFP is measured at the opposite pole of the cell relative to the synaptic input. Restorative conductances are typically expressed in the LFP as resonance peaks. However, these are considerably smaller than the corresponding peaks for the membrane potentials and transmembrane currents.

We compared the findings from our quasi-active models with published, experimentally constrained pyramidal cell models that display various active conductances. We found that in particular the hyperpolarization-activated inward current, I_H_, can have a sizable impact on the shape of the LFP, since it is a restorative current, strong in the apical dendrite, and active at subthreshold voltages.

We conclude that subthreshold contributions of active currents to the LFP of detailed pyramidal cell models can be very well captured by quasi-active models containing one or two voltage-dependent currents, and that their contribution can be a major factor in shaping the LFP for the lowest frequencies.

